# Macroalgae and interspecific alarm cues regulate behavioral interactions between sea urchins and sea cucumbers

**DOI:** 10.1038/s41598-022-07889-8

**Published:** 2022-03-10

**Authors:** Jiangnan Sun, Yushi Yu, Zihe Zhao, Ruihuan Tian, Xiang Li, Yaqing Chang, Chong Zhao

**Affiliations:** 1 Key Laboratory of Mariculture and Stock Enhancement in North China’s Sea, Ministry of Agriculture and Rural Affairs, Dalian Ocean University, Dalian, 116023 China; 2 Southern Marine Science and Engineering Guangdong Laboratory, Guangzhou, 511458 China

**Keywords:** Behavioural ecology, Animal behaviour

## Abstract

Sea urchins and sea cucumbers are mutually beneficial organisms in kelp ecosystem. As herbivores, sea urchins process kelp through feeding and egestion, providing inaccessible food for benthic consumers such as sea cucumbers. Sea urchins in turn profit from the sediment cleaned by sea cucumbers. However, behavioral interactions between them remain poorly understood, which greatly hampers our understanding on the relationship between ecologically important benthic species in marine ecosystems and the regulating mechanism. The present study investigated behavioral interactions between sea urchins *Strongylocentrotus intermedius* and sea cucumbers *Apostichopus japonicus* in laboratory conditions. We revealed that the presence of sea urchins caused significant higher speed movement of *A. japonicus*. Interestingly, the negative effects of *S. intermedius* on *A. japonicus* were significantly reduced in the shared macroalgal area. For the first time, we found the interspecific responses to alarm cues between sea cucumbers and sea urchins. Conspecific responses were significantly larger than the interspecific responses in both sea urchins and sea cucumbers. This indicates that interspecific response to alarm cues is an efficient approach to anti-predation and coexistence in mutually beneficial organisms. The present study shed light on the interspecific relationships and coexistence between sea urchins and sea cucumbers in kelp ecosystem.

## Introduction

Sea urchins and sea cucumbers are ecologically important benthic organisms in kelp ecosystems^1,2^. Sea urchins, as herbivores, convert the coarse particulate organic matter of kelp into fecal particles that benefit marine scavengers, including sea cucumbers^3,4^. Sea urchins in turn profit from the bioremediation of sea cucumbers^5^. The mutual benefits between sea urchins and sea cucumbers enhance the nutrient cycling in benthic communities and improve the productivity of kelp ecosystems^3,5^. Despites their mutually beneficial relationship in kelp ecosystems, the two species are not highly compatible. Previous study reported cases of sea urchins *Strongylocentrotus droebachiensis* preying on small sea cucumbers *Cucumaria frondosa*^6^. Potentially negative interspecific relationships affect the local distribution of these two ecologically important organisms, while the mutual benefits highly depend on their coexistence^7^. Therefore, it is important to investigate the behavioral interactions between sea urchins and sea cucumbers and the ecological consequence.

The absence of functionally important organisms on basal trophic levels, however, triggers cascading effects on kelp ecosystems that might weaken their ability to withstand other large-scale stressors^8,9^. It is thus important to investigate what factors regulate their behavioral interactions that allow them to coexist in kelp ecosystems. Macroalgae are foundation species in the kelp ecosystem and important resources^10^. By providing food and habitats, macroalgae enhance the biodiversity of kelp ecosystem and support the interactions among various species^11,12^. Sea urchins and sea cucumbers well coexist in benthic communities with abundant macroalgal biomass^13–15^. Overgrazing of kelp by sea urchins, however, destroyed the habitat of other benthic organisms and reduced local biodiversity and biomass^16^. But this negative effect was reduced in macroalgal-rich communities^17^. We thus hypothesized that macroalgae probably regulate the interactions between sea urchins and sea cucumbers and contribute to their coexistence, besides from providing organic matters. Predator avoidance operate as selective forces leading to a positive relationship between benthic organisms in marine ecosystems^18^. Joint anti-predation strategy is probably an important approach for the mutualism between sea urchins and sea cucumbers and contributes to their stable existence in benthic communities. In the kelp ecosystem, sea urchins and sea cucumbers are threatened by similar predators, including starfish^19,20^, crustaceans^21,22^ and fishes^23^. Rapid behavioral responses to alarm cues from injured or killed conspecifics can avoid the risk of predation^24^. Sea urchins are well documented to respond to alarm cues from injured or killed conspecifics^25–28^. However, it remains unknown whether sea cucumbers respond to conspecific and interspecific alarm cues. Sharing alarm signals or joint anti-predation is a common approach for mutual benefits^29,30^. Thus, we hypothesized that interspecific alarm cues exist as mutual benefits between sea urchins and sea cucumbers, because of their coexistence and the exposure to similar predators in kelp ecosystem.

The sea urchin *Strongylocentrotus intermedius* and the sea cucumber *Apostichopus japonicus* coexist in coastal kelp ecosystems in the coastal areas of Northeast Asia^31–33^. Behavioral responses to environmental changes and alarm cues have been well documented in *S. intermedius*^28,34,35^. In addition, *S. intermedius* and *A. japonicus* are in exposure to similar predators, including *Charybdis japonica*^36^ and *Asterina pectinife*^37,38^. Therefore, *S. intermedius* and *A. japonicus* are good research models to investigate interactions between sea urchins and sea cucumbers. The main purposes of the present study are to investigate: (1) whether behavioral interactions exist between sea urchins and sea cucumbers; (2) whether macroalgae regulate the interactions between sea urchins and sea cucumbers; (3) whether sea cucumbers respond to the conspecific alarm cues; (4) whether there are interspecific responses to alarm cues between sea urchins and sea cucumbers.

## Results

### Behavioral interactions exist between sea urchins and sea cucumbers

**Figure 1:**
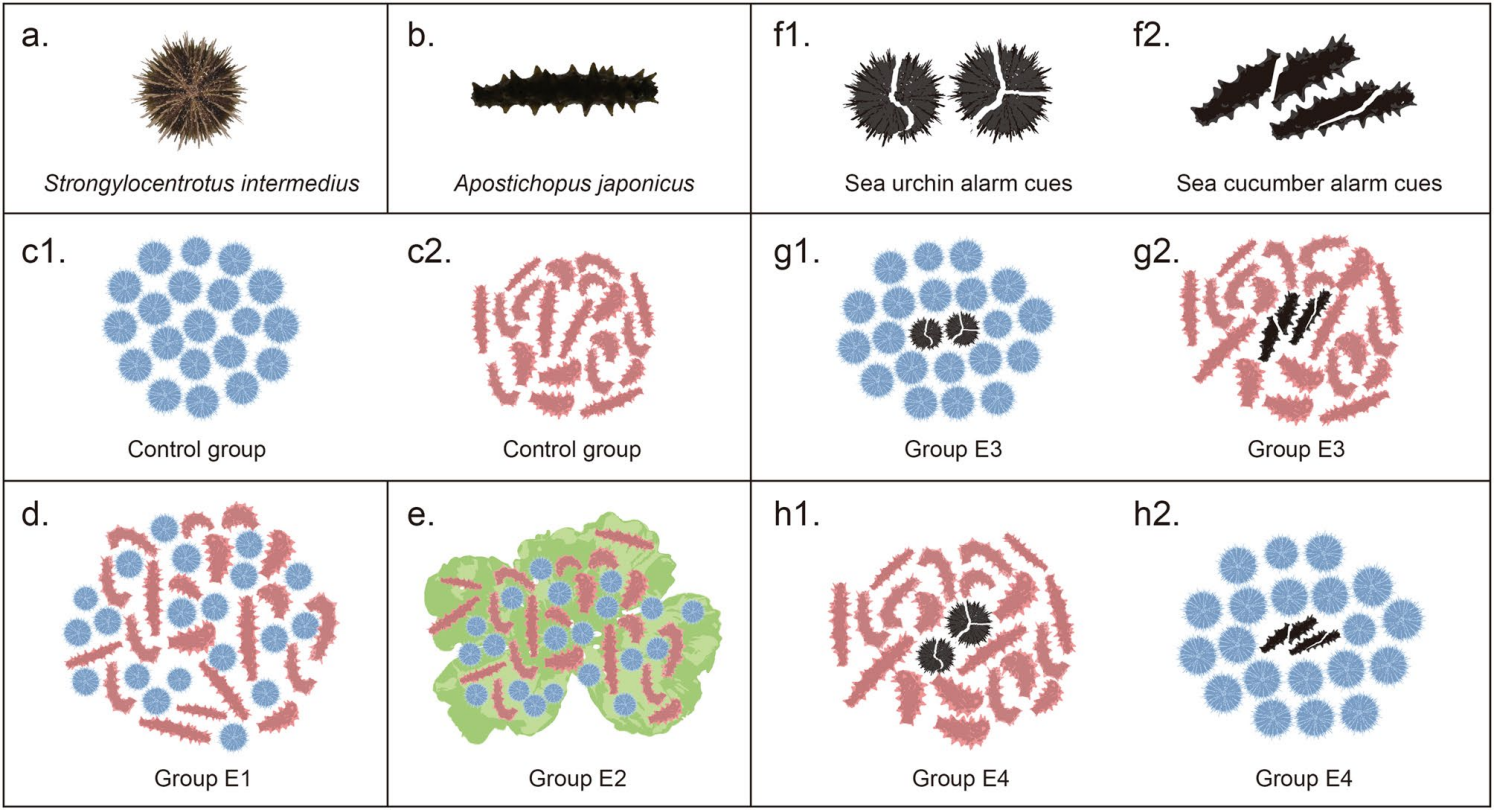
Schematic diagrams for the experiments. (**a**) *Strongylocentrotus intermedius* and (**b**) *Apostichopus japonicus*. In experiment 1, either 20 sea urchins or 20 sea cucumbers in different tanks were recorded as the control group (**c**). Twenty sea urchins and 20 sea cucumbers in a tank were recorded as group E1 (**d**). In experiment 2, 20 sea urchins and 20 sea cucumbers in a tank with macroalgae *Ulva lactuca* were recorded as group E2 (**e**). Two injured *S. intermedius* and two injured *A. japonicus* were used as the source of alarm cues (**f**). In experiment 3, the behavioral responses to conspecific alarm cues of sea urchins and sea cucumbers were recorded as group E3 (**g**). The behavioral response to interspecific alarm cues of sea urchins and sea cucumbers were recorded as group E4 (**h**).

**Figure 2:**
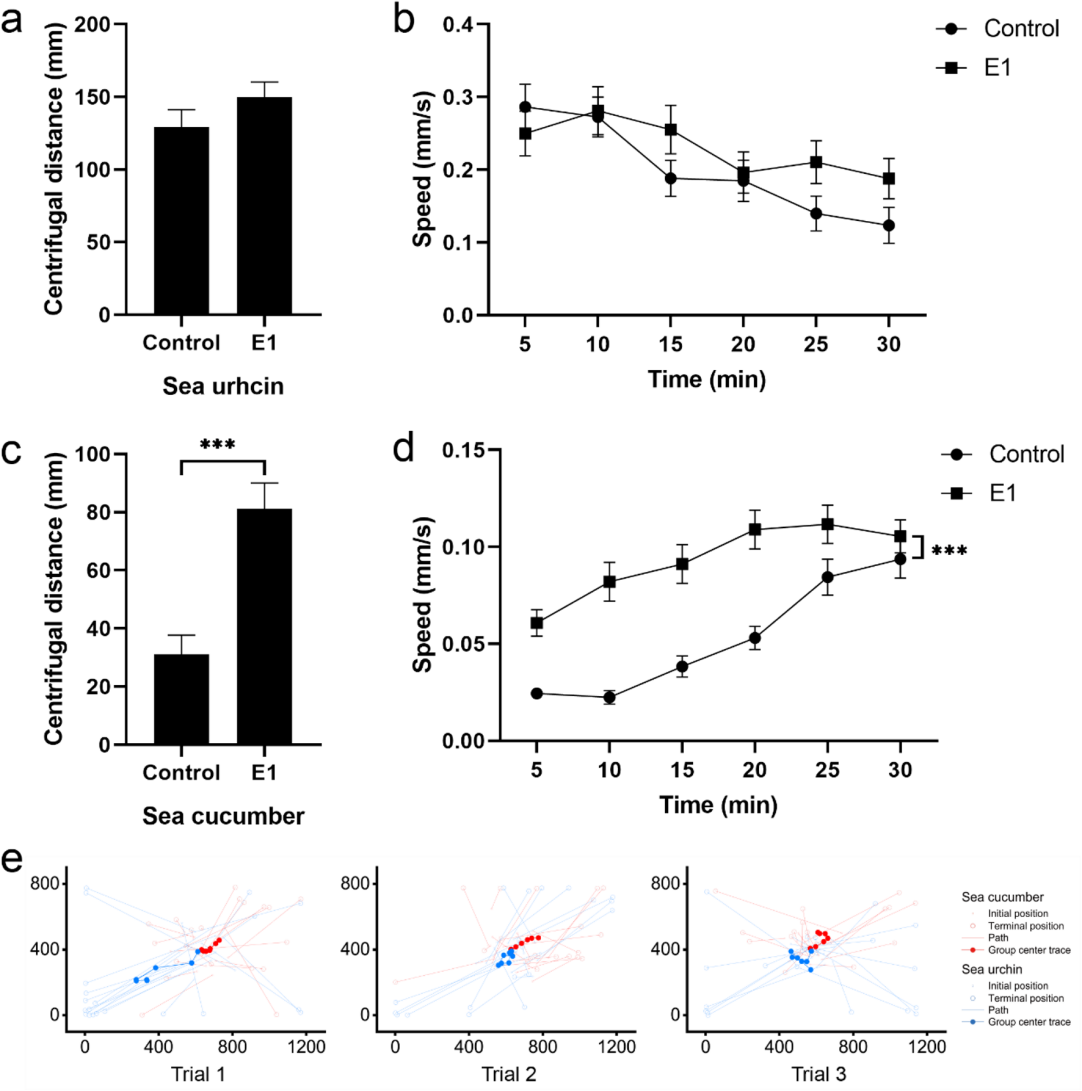
Behavioral interactions exist between sea urchins and sea cucumbers. (**a**) Average centrifugal distance and (**b**) movement speed (mean ± SEM) of sea urchins in the control group and group E1. (**c**) Average centrifugal distance and (**d**) movement speed (mean ± SEM) of sea cucumbers in control group and group E1. (**e**) The initial positions (small hollow point) and terminal positions (large hollow point) of sea cucumbers (light red) and sea urchins (light blue). Tracking of the center position (line through solid points) of the sea cucumber group (bright red) and the sea urchin group (bright blue) in all the three trials.

To investigate whether behavioral interactions exist between sea urchins and sea cucumbers, 20 sea urchins *S. intermedius* (Fig. 1a) and 20 sea cucumbers *A. japonicus* (Fig. 1b) were put in a tank for group E1 (Fig. 1d), comparing 20 sea urchins and 20 sea cucumbers of control group (Fig. 1c). Experiment of each group were repeated three times. Individual behaviors of all 60 animals in each experiment were compared between control group and group E1 (n = 60). The presence of sea cucumbers did not significantly affect the centrifugal distance (control group: 129.18 ± 12.05 mm; group E1: 149.60 ± 10.57 mm, *P* = 0.332, Fig. 2a) and movement speed of sea urchins (S1, *P* = 0.227, Fig. 2b). However, sea urchins significantly increased the centrifugal distance (control group: 31.05 ± 6.62 mm; group E1: 81.15 ± 8.90 mm, *P* < 0.001, Fig. 2c) and movement speed of sea cucumbers (S2, *P* < 0.001, Fig. 2d). The movement tracks of group center of sea urchins and sea cucumbers did not overlap and moved in opposite directions in all three trials of group E1 (Fig. 2e).

### Macroalgae regulate the interaction between sea urchins and sea cucumbers

**Figure 3:**
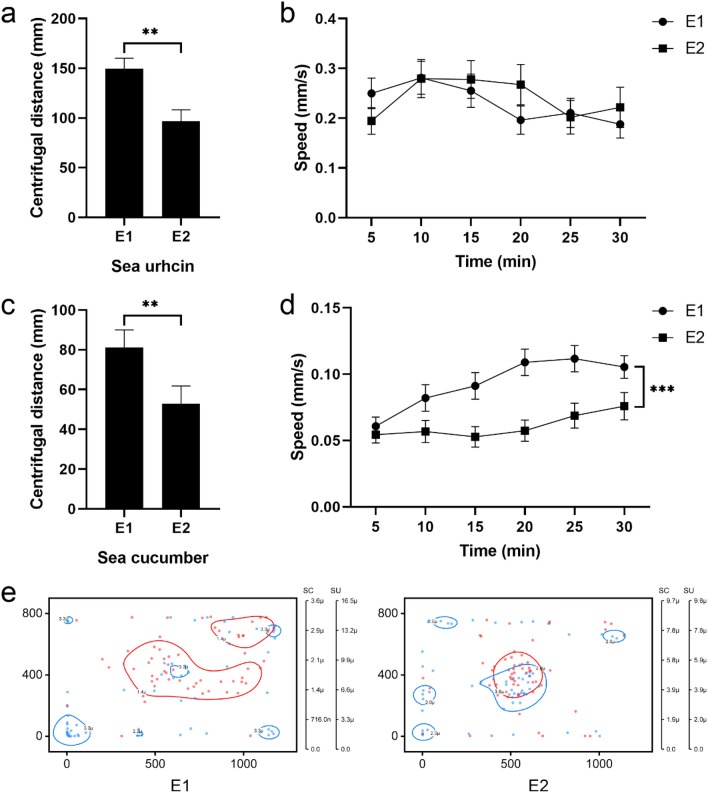
Macroalgae regulate the interactions between sea urchins and sea cucumbers. (**a**) Average centrifugal distance and (**b**) movement speed (mean ± SEM) of sea urchins in groups E1 and E2. (**c**) Average centrifugal distance and (**d**) movement speed (mean ± SEM) of sea cucumbers in groups E1 and E2. (**e**) The position of sea urchins (blue points) and sea cucumbers (red points) in groups E1 and E2.

To investigate whether macroalgae regulate the interactions between sea urchins and sea cucumbers, *Ulva lactuca* was put in the center of the tank with 20 sea urchins and 20 sea cucumbers for group E2 (Fig. 1e). Experiment of group E2 were repeated three times (n = 60). In the presence of *U. lactuca*, the centrifugal distance of sea urchins decreased significantly (group E2: 96.92 ± 11.30 mm, *P* = 0.001, Fig. 3a), but the movement speed of sea urchins did not change significantly (S1, *P* = 0.770, Fig. 3b). The presence of *U. lactuca* significantly reduced centrifugal distance (group E2: 52.84 ± 8.94 mm, *P* = 0.004, Fig. 3c) and movement speed of sea cucumbers (S2, *P* < 0.001, Fig. 3d). At the end of the experiment 2, the sea urchins and sea cucumbers mainly distributed in the position of macroalgae (kernel density estimation in group E2: sea urchin > 2.0 µ, sea cucumber > 3.9 µ, Fig. 3e), while sea cucumbers mainly distributed in the middle of the tank and sea urchins mainly distributed on the edges without macroalgae (kernel density estimation in group E1: sea urchin > 3.3 µ, sea cucumber > 1.4 µ, Fig. 3e).

### Sea urchins and sea cucumbers respond to the conspecific and interspecific alarm cues

**Figure 4:**
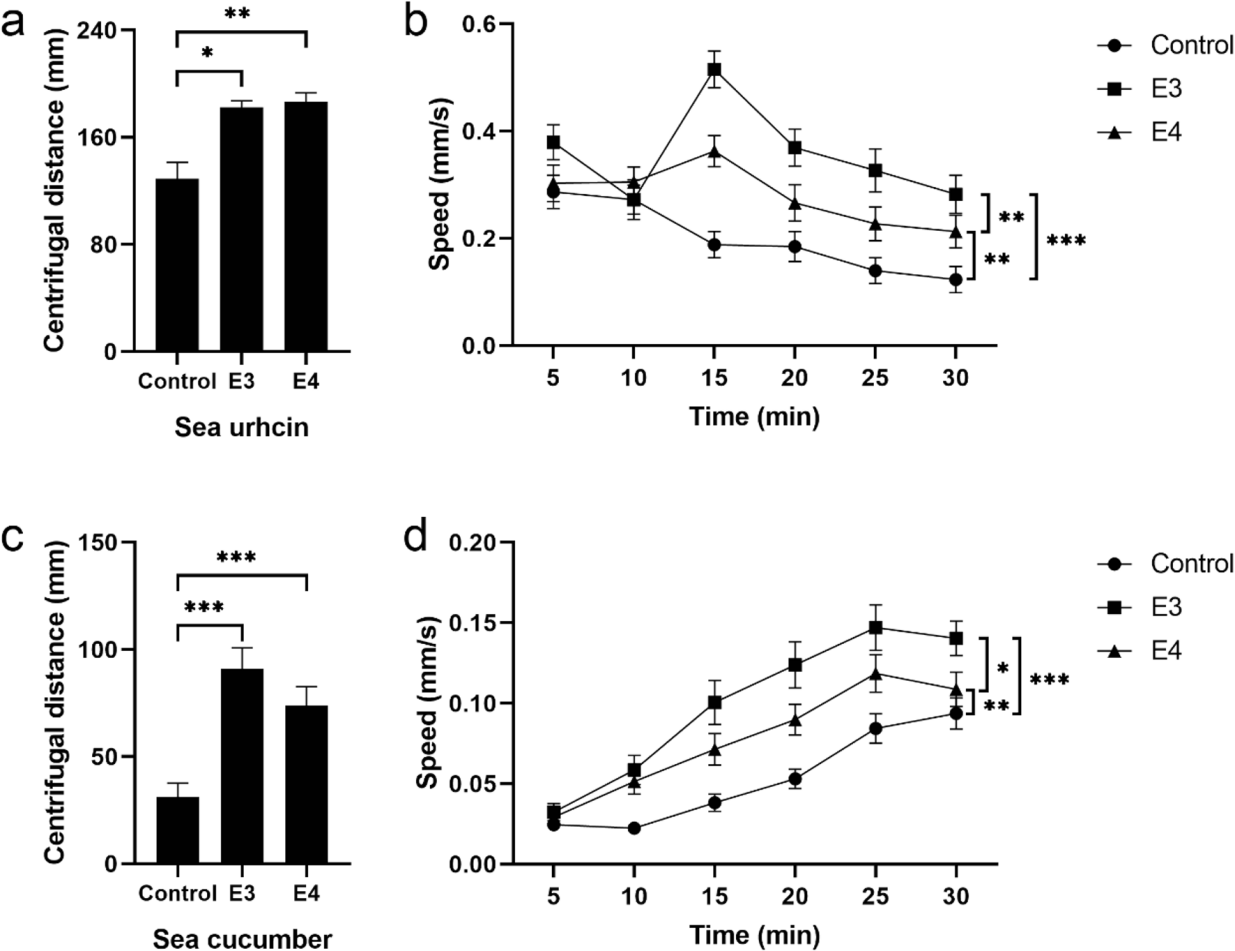
Sea urchins and sea cucumbers respond to the conspecific and interspecific alarm cues. (**a**) Average centrifugal distance and (**b**) movement speed (mean ± SEM) of sea urchins in the control group and group E3 and E4. (**c**) Average centrifugal distance and (**d**) movement speed (mean ± SEM) of sea cucumbers in the control group and groups E3 and E4.

Two injured sea urchins or sea cucumbers were put in the center of the tank as the source of alarm cues (Fig. 1f) with 20 conspecifics around for group E3 (Fig. 1g). Experiment of group E3 were repeated three times (n = 60). Conspecific alarm cues significantly increased centrifugal distance (group E3: 182.29 ± 5.05 mm, *P* = 0.017, Fig. 4a) and movement speed of sea urchins (S1, *P* < 0.001, Fig. 4b). The centrifugal distance of sea cucumbers exposed to the conspecific alarm cues was significantly higher than that of sea cucumbers in the control group (group E3: 90.99 ± 9.80 mm, *P* < 0.001, Fig. 4c). Sea cucumber alarm cues significantly increased the movement speed of sea cucumbers (S2, *P* < 0.001, Fig. 4d).

Two injured sea urchins and sea cucumbers were separately put in the center of the tank as the source of alarm cues with 20 sea cucumbers and 20 sea urchins around respectively (group E4, Fig. 1h). Experiment of group E4 were repeated three times (n = 60). The centrifugal distance of sea urchins exposed to sea cucumber alarm cues was significantly higher than that of sea urchins in the control group (group E4: 186.36 ± 6.87 mm, *P* = 0.001, Fig. 4a). The movement speed of sea urchins in group E4 was significantly higher than that of sea urchins in the control group (S1, *P* = 0.002, Fig. 4b). Sea urchin alarm cues significantly increased the centrifugal distance (group E4: 73.94 ± 8.76 mm, *P* < 0.001, Fig. 4c) and movement speed of sea cucumbers (S2, *P* = 0.008, Fig. 4d). Interestingly, the movement speed of sea urchins exposed to sea urchin alarm cues was significantly higher than that of sea urchins exposed to sea cucumber alarm cues (S1, *P* = 0.003, Fig. 4b). The movement speed of sea cucumbers exposed to sea cucumber alarm cues was significantly higher than that of sea cucumbers exposed to sea urchin alarm cues (S2, *P* = 0.020, Fig. 4d).

## Discussion

The present study revealed the behavioral interactions between sea urchins *S. intermedius* and sea cucumbers *A. japonicus*. The presence of sea urchins caused significantly higher speed movement of sea cucumbers. Physical contact with sea urchins cause sea cucumbers to flee, because the spines of sea urchins are highly irritating^39,40^. This explains the dominance of sea urchins in benthic communities, not only in competition for food, but for more habits. Therefore, sea urchins in the wild would easily form dominant populations, which makes it difficult for kelp beds (or forests) to recover from barrens without anthropogenic culling urchins or introducing predators^41,42^. Further, the negative effects of sea urchins on important functional organisms such as sea cucumbers, probably impact their coexistence in kelp ecosystems and eventually weaken the trophic cascade of systems, besides from the grazing of kelp. The present study improves our understanding of potential competition or negative interspecific relationships between sea urchins and sea cucumbers, in addition to mutual benefits.

The regulation of behavioral interactions between the ecologically important organisms is thus important in the kelp ecosystem. Negative behavioral interaction does not affect the coexistence of sea urchins and sea cucumbers in benthic communities with abundant macroalgal biomass, where sea urchins and sea cucumbers share the habitats^14,15^. Macroalgae enhance the biodiversity of kelp ecosystem by providing food and habitat^10–12^. The function of sea urchins to change the availability of habitat is important for attracting mobile organisms to the kelp ecosystem^43,44^. Unlike the group without macroalgae, sea cucumbers were kept in the macroalgal area and well coexisted with sea urchins in the present study. This novel finding indicates that macroalgae greatly reduce the negative effect of sea urchins in causing the higher speed movement of sea cucumbers. The mutual benefit between sea urchins and sea cucumbers thus highly depends on the presence of macroalgae^3,5^. Restoration of kelp communities is of great importance to marine ecosystem, because of a variety of ecological functional roles of macroalgae^45,46^. The present study reveals the important regulating function of macroalgae in the interspecific relationships between ecologically important organisms and highlights the importance of macroalgae in kelp community management.

Response to alarm cues is an important method for anti-predation of benthic organisms in the kelp ecosystem^18^. The behavior response to alarm cues has been well documented in sea urchins^25–28^. Unsurprisingly, *S. intermedius* quickly moved away from the source of alarm cues. This strategy effectively reduced the risk of predation on sea urchins^24^. For the first time, we revealed that *A. japonicus* showed escaping behavior to the alarm cues from injured conspecifics. Detecting cues and subsequent escaping is a cost-effective strategy, compared to the other defense methods of sea cucumbers, such as burying and spitting out internal organs^5^. Joint anti-predation is mutually benefit for mutualists^30^. Interspecific responses to alarm cues were reported between sea urchin species, for example *S. intermedius* and *Mesocentrotus nudus*^28^. The response to interspecific alarm cues between sea urchins and sea cucumbers is totally unknown, despites the long coexistence and exposure to the same predators in kelp ecosystem. In the present study, injured *A. japonicus* caused significantly higher speed movement of *S. intermedius*, while healthy sea cucumbers had no significant effect on the behavior of sea urchins. Consistently, sea cucumbers respond to the alarm cues from injured sea urchins and moved away from the cues source. This suggests the behavioral responses to interspecific alarm cues between sea urchins and sea cucumbers. Detecting alarm cues from other species help sea urchins and sea cucumbers make a strategy in advance. Interestingly, the effect of the interspecific alarm cues was significantly weaker than that of conspecific alarm cues. This clearly indicates that joint anti-predation strategies exist between sea urchins and sea cucumbers, besides from behavioral responses to chemical stimuli^47^. The response of sea urchins and sea cucumbers to interspecific alarm cues is important for the mutualism between these two ecologically important organisms and contributes to their coexistence in kelp ecosystems. This sheds light on the potential mutual benefits between benthic organisms exposed to predation stress in kelp ecosystems.

The present study reveals the negative behavioral interactions between sea urchins and sea cucumbers. This indicates that macroalgae play an important role in regulating interspecific relationships between these benthic organisms and in supporting their mutualism in kelp ecosystems. Further, responding to interspecific alarm cues is an important approach for joint anti-predation of invertebrates in marine benthic communities and contributes to their coexistence in kelp ecosystems. Notably, field studies are essential to further test the present laboratory investigation in future.

## Methods

### Animals

The sea urchins (~ 0.8 g of wet body weight, Fig. 1a) and sea cucumbers (~ 0.6 g of wet body weight, Fig. 1b) were transported from hatcheries to the Key Laboratory of Mariculture and Stock Enhancement in North China's Sea, Ministry of Agriculture and Rural Affairs at Dalian Ocean University (121° 37ʹ E, 38° 87ʹ N). Sea urchins were maintained at 10 ± 0.5 °C in a 300 L tank in the laboratory. During this period, we fed sea urchins with fresh macroalgae *Ulva lactuca* at night every day and cleaned the residual food and feces in the tank. One third of the water in the tank was replaced every two days. The sea cucumbers were kept in a 500 L tank at 10 ± 0.5 °C, fed a commercial diet (Anyuan Industrial Co., Ltd)^48^. We cleaned up the feces of sea cucumbers every day and changed one-third of the seawater every two days. All experiments were carried out in laboratory at low light intensity of ~ 20 lx^49^.

### Experimental design

#### Experiment 1: whether behavioral interactions exist between sea urchins and sea cucumbers

The behaviors were recorded for 20 sea urchins without external stimuli in a tank (length × width × height: 420 × 280 × 250 mm) and 20 sea cucumbers in the other tank (control group, Fig. 1c). To investigate whether behavioral interactions exist between sea urchins and sea cucumbers, we put 20 sea urchins and 20 sea cucumbers in a tank (group E1, Fig. 1d) and tracked their locations and movement through the video of the experiment using Manual Tracking plugin for ImageJ software (version 1.51n). At the beginning of the experiment, sea cucumbers and sea urchins were randomly placed in the center of the tank to ensure the random distribution among them (Fig. 1d). Experiments of each group were repeated three times using different sea urchins and sea cucumbers for the control group (n = 60) and group E1 (n = 60).

#### Experiment 2: whether macroalgae regulate the interactions between sea urchins and sea cucumbers

*Ulva lactuca* is a common macroalgal species in the habitats of *S. intermedius* and *A. japonicus*^50,51^. To investigate whether macroalgae regulate the interactions between sea urchins and sea cucumbers, we placed the *U. lactuca* in the center of the tank with a small stone and repeated the measurements in experiment 1 (group E2, Fig. 1e). At the beginning of the experiment, 20 sea cucumbers and 20 sea urchins were randomly placed on the macroalgae *Ulva lactuca*. The locations and movements of sea urchins and sea cucumbers were recorded and tracked using ImageJ software (version 1.51n). The experiment was repeated three times using different sea urchins and sea cucumbers (n = 60).

#### Experiment 3: whether sea urchins and sea cucumbers respond to the conspecific and interspecific alarm cues

The body of injured animal is a common signal source of alarm cues in previous studies^28^. In the present study, two injured *S. intermedius* and two injured *A. japonicus* were used as the source of alarm cues (Fig. 1f). To investigate the behavioral response to conspecific alarm cues, we placed two injured sea urchins or sea cucumbers in the center of the tank with 20 conspecifics around (group E3, Fig. 1g). To investigate whether there are interspecific responses to alarm cues, we exchanged the source of the alarm cues and repeated the behavioral experiments (group E4, Fig. 1h). The locations and movements of sea urchins and sea cucumbers were recorded. Experiments of each group were repeated three times using different sea urchins and sea cucumbers for the group E3 (n = 60) and group E4 (n = 60).

For each trial, the arena tank (420 × 280 × 250 mm) was filled to a depth of 60 mm with fresh seawater. Above the tank, we placed a Canon HF20 digital video camera, which took time-lapse pictures of the entire tank (3840 × 2160 pixels, 30 s time-lapse shot). The experiment began when all the animals were placed in the center of the tank. All experiments lasted 30 min. The seawater was changed for each trial to avoid potential non-experimental impacts.

### Movement speed and centrifugal movement distance

Movement speed is an important index to study the behavioral response of sea urchins and sea cucumbers^38–40^. To calculate the average speed of the experimental animals, we extracted the coordinates of each animal every five minutes by using ImageJ (version 1.51n). The movement speed (*v*) of the animal was calculated as follows:

$$\begin{array}{c}{v}_{i}=\sqrt{{\left[{x}_{i}\left(t\right)-{x}_{i}\left(t-5\right)\right]}^{2}+{\left[{y}_{i}\left(t\right)-{y}_{i}\left(t-5\right)\right]}^{2}}\times k/300\end{array}$$

where (*x*_*i*_(*t*)*, y*_*i*_(*t*)) is the coordinates of the animal *i* at minute *t*,* k* is the scale of the picture.

Spreading out from the aggregations is an important behavioral process for sea urchins and sea cucumbers to reduce the competition within groups^52,53^. We thus calculated the distance of the animals from the center of the tank at the beginning and the end of the experiment to analyze their centrifugal movement. The average centrifugal distance (*d*) was calculated as follows:

$$\begin{array}{c}d=\sum (\sqrt{{\left[{x}_{i}\left(30\right)-{x}_{t}\right]}^{2}+{\left[{y}_{i}\left(30\right)-{y}_{t}\right]}^{2}}-\sqrt{{\left[{x}_{i}\left(0\right)-{x}_{t}\right]}^{2}+{\left[{y}_{i}\left(0\right)-{y}_{t}\right]}^{2}})\times k/20\end{array}$$

where (*x*_*i*_(30)*, y*_*i*_(30)) is the coordinates of the animal *i* at the end of the experiment, (*x*_*t*_*, y*_*t*_) is the coordinates of the center of the tank, (*x*_*i*_(0)*, y*_*i*_(0)) is the coordinates of the animal *i* at the beginning of the experiment,* k* is the scale of the picture.

In order to compare the distribution changes, we calculated the location of the group center every five minutes in both sea urchin and sea cucumber groups. The coordinates (*x*_*c*_*, y*_*c*_) of the group center were the average of the coordinates of all animals in the group:

$$\begin{array}{c}({x}_{c}\left(t\right),{y}_{c}\left(t\right))=(\sum {x}_{i}\left(t\right),\sum {y}_{i}\left(t\right))/20\end{array}$$

where (*x*_*c*_(t)*, y*_*c*_(t)) is the coordinates of the group center at minute *t*, (*x*_*i*_(*t*)*, y*_*i*_(*t*)) is the coordinates of the animal *i* in the group at minute *t*.

In order to intuitively describe the distributions of sea urchins and sea cucumbers, 2D Kernel Density plot was drawn according to the locations of animals at the end of the experiment using OriginPro 2019b (version 9.6.5)^54^.

### Statistical analysis

The data were tested for homogeneity of variance and normal distribution before all statistical analyses using the Levene test and Shapiro–Wilk test, respectively. In experiment 1, Mann–Whitney U test was used to compare the centrifugal distance of experimental animals between the control group and group E1. One-way repeated measures ANOVA was used to compare the movement speeds of the animals between the two groups in experiment 1. Least-Significant Difference was used for the following post hoc test. In experiment 2, the centrifugal distance was compared by using Mann–Whitney U test in groups E1 and E2. One-way repeated measures ANOVA and Least-Significant Difference were used to compare the movement speeds of animals in the two groups in experiment 2. To investigate whether there are interspecific responses to alarm cues between sea urchins and sea cucumbers, the centrifugal distance of animals was compared among the control group and groups E3 and E4 by using Mann–Whitney U test. One-way repeated measures ANOVA was used to compare the movement speeds between the three groups and Least-Significant Difference were subsequently used for post hoc test in experiment 3.

### Ethical approval

All applicable international, national, and/or institutional guidelines for the care and use of animals were followed by the authors.

## Supplementary Information

Supplementary Information.

## Data Availability

All data generated or analyzed during this study are included in this published article (and its Supplementary Information files S1).
