# Supplementary material for: Macroalgae and interspecific alarm cues regulate behavioral interactions between sea urchins and sea cucumbers
Source: Sci Rep. 2022 Mar 10;12:3971. doi: 10.1038/s41598-022-07889-8 (PMC8913812; doi:10.1038/s41598-022-07889-8)
Supplement: Supplementary file 1 — Supplementary Information. [file 41598_2022_7889_MOESM1_ESM.docx]

**Supplementary information for**

**Macroalgae and interspecific alarm cues regulate behavioral interactions between sea urchins and sea cucumbers**

Jiangnan Sun^1^, Yushi Yu^1^, Zihe Zhao^1^, Ruihuan Tian^1^, Xiang Li^1^, Yaqing Chang^1*^, Chong Zhao^1,2*^

^1^ Key Laboratory of Mariculture & Stock Enhancement in North China's Sea, Ministry of Agriculture and Rural Affairs, Dalian Ocean University, Dalian 116023, China.

^2^ Southern Marine Science and Engineering Guangdong Laboratory, Guangzhou，511458, China

^*^email: chongzhao@dlou.edu.cn (C Zhao), changlab@hotmail.com (Y Chang)

**Supplementary Table 1**

**Data of movement speed.** Data of movement speed (mm/s) of sea urchins every five minutes in control group and groups E1, E2, E3 and E4 (mean ± SEM).

| Time (min) | Control group | Group E1 | Group E2 | Group E3 | Group E4 |
| --- | --- | --- | --- | --- | --- |
| 5 | 0.29 ± 0.03 | 0.25 ± 0.03 | 0.19 ± 0.03 | 0.38 ± 0.03 | 0.30 ± 0.03 |
| 10 | 0.27 ± 0.03 | 0.28 ± 0.03 | 0.28 ± 0.04 | 0.27 ± 0.04 | 0.31 ± 0.03 |
| 15 | 0.19 ± 0.02 | 0.26 ± 0.03 | 0.28 ± 0.04 | 0.52 ± 0.03 | 0.36 ± 0.03 |
| 20 | 0.18 ± 0.03 | 0.20 ± 0.03 | 0.27 ± 0.04 | 0.37 ± 0.03 | 0.27 ± 0.03 |
| 25 | 0.14 ± 0.02 | 0.21 ± 0.03 | 0.20 ± 0.03 | 0.33 ± 0.04 | 0.23 ± 0.03 |
| 30 | 0.12 ± 0.02 | 0.19 ± 0.03 | 0.22 ± 0.04 | 0.28 ± 0.04 | 0.21 ± 0.03 |

**Supplementary Table 2**

**Data of movement speed.** Data of movement speed (mm/s) of sea cucumbers every five minutes in control group and groups E1, E2, E3 and E4 (mean ± SEM).

| Time (min) | Control group | Group E1 | Group E2 | Group E3 | Group E4 |
| --- | --- | --- | --- | --- | --- |
| 5 | 0.025 ± 0.003 | 0.061 ± 0.007 | 0.055 ± 0.006 | 0.032 ± 0.005 | 0.029 ± 0.004 |
| 10 | 0.022 ± 0.004 | 0.082 ± 0.010 | 0.057 ± 0.008 | 0.059 ± 0.009 | 0.052 ± 0.008 |
| 15 | 0.038 ± 0.005 | 0.091 ± 0.010 | 0.053 ± 0.008 | 0.101 ± 0.014 | 0.072 ± 0.010 |
| 20 | 0.053 ± 0.006 | 0.109 ± 0.010 | 0.058 ± 0.008 | 0.124 ± 0.014 | 0.090 ± 0.010 |
| 25 | 0.084 ± 0.009 | 0.112 ± 0.010 | 0.069 ± 0.009 | 0.147 ± 0.014 | 0.119 ± 0.012 |
| 30 | 0.094 ± 0.010 | 0.105 ± 0.009 | 0.076 ± 0.010 | 0.140 ± 0.011 | 0.109 ± 0.011 |

**Supplementary Table 3**

**Data of centrifugal distance.** Data of centrifugal distance (mm) of sea urchins and sea cucumbers in control group and groups E1, E2, E3 and E4 (mean ± SEM).

|  | Control group | Group E1 | Group E2 | Group E3 | Group E4 |
| --- | --- | --- | --- | --- | --- |
| Sea urchins | 129.18 ± 12.05 | 149.60 ± 10.57 | 96.92 ± 11.30 | 182.29 ± 5.05 | 186.36 ± 6.87 |
| Sea cucumbers | 31.05 ± 6.62 | 81.15 ± 8.90 | 52.84 ± 8.94 | 90.99 ± 9.80 | 73.94 ± 8.76 |
